# Pulse-by-pulse transient thermal deformation in crystal optics under high-repetition-rate FEL

**DOI:** 10.1107/S160057752401155X

**Published:** 2025-01-01

**Authors:** Lin Zhang, Jerome Hastings, Zhirong Huang, Jingyi Tang, Diling Zhu

**Affiliations:** a LCLS SLAC National Accelerator Laboratory 2575 Sand Hill Road Menlo Park CA94025 USA; Australian Synchrotron, Australia

**Keywords:** cavity-based free-electron laser, X-ray crystal optics, pulse-by-pulse transient thermal deformation, pulse energy, repetition rate, first-turn time, period-end time

## Abstract

Time-domain modeling of the thermal deformation of crystal optics can help define acceptable operational ranges across the pulse-energy repetition-rate phase space. In this paper, we have studied the transient thermal deformation of a water-cooled diamond crystal for a cavity-based X-ray free-electron laser (CBXFEL), either an X-ray free-electron laser oscillator (XFELO) or a regenerative amplifier X-ray free-electron laser (RAFEL), by numerical simulations including finite-element analysis and advanced data processing. Pulse-by-pulse transient thermal deformation of a 50 µm-thick diamond crystal has been performed with X-ray pulse repetition rates between 50 kHz and 1 MHz. Results for temperature and thermal deformation have been compared with the results of transient analysis using a continuous wave (CW) power loading. Temperature and thermal deformation results from pulse-by-pulse transient analysis vary with time about the results for the CW case for the same average power. The variation amplitude increases with pulse energy and decreases with repetition rate. When the repetition rate increases to infinity, both temperature and thermal deformation converge to the results for the CW case. Two critical time scales for the operation of crystal optics in a CBXFEL are (1) first-turn time, *i.e.* the time for the XFEL pulse to complete the first turn around the cavity so that the crystal sees the recirculated XFEL pulse, and (2) period-end time, *i.e.* the time that the next electron bunch arrives for the amplification, so that the crystal outcouples the amplified FEL power. For the same average power, simulation results show that the crystal thermal deformation seen by the XFEL beam decreases with repetition rate at the first-turn time of a 300 m-long cavity and increases with repetition rate at the period-end time. For the wavefront preservation requirement of the crystal optics, a pulse-energy versus repetition-rate phase space has been established. The upper bounds of the pulse energy at both first-turn and period-end times decreases with repetition rate, especially at the period-end time. The upper bound of the thermal deformation of the crystal at the period-end time for any repetition frequency can be estimated from the CW case. For a water-cooled diamond crystal of dimension 5 mm × 5 mm × 0.05 mm, the time to reach a quasi steady-state is about 50 ms for temperature and 50 µs for thermal deformation

## Abstract 2

Complete pulse-by-pulse transient time-domain modeling of the thermal deformation of crystals under high-repetition-rate high-pulse-energy free-electron laser load can help define acceptable operational parameters across the pulse-energy repetition-rate phase space. The upper bound of the thermal deformation of the crystal at the electron beam repetition time for any repetition frequency can be estimated from the results of transient analysis using a continuous wave power loading.

## 1. Introduction

Hard X-ray free-electron laser (FEL) sources deliver an X-ray beam with outstanding properties such as fs-scale pulse lengths, mJ-scale pulse energy, and near full transverse coherence. High-power FEL pulses can produce a significant spike in temperature, thermal deformation and elastic waves in X-ray optics. The elastic waves can superimpose on the temperature gradient induced thermal deformation. For the low-repetition-rate FELs, such as LCLS (Emma *et al.*, 2010 ▸), SACLA (Ishikawa *et al.*, 2012 ▸), SwissFEL (Prat *et al.*, 2020 ▸) and PAL-XFEL (Kang *et al.*, 2017 ▸), the repetition time is equal to or greater than 8.33 ms. This time is long enough for the X-ray optics to recover from the spikes in thermal deformation and operate with stable performance from the previous pulse. High-repetition-rate FELs are emerging around the world. The European XFEL (EuXFEL) is operational offering bursts of MHz hard X-rays (Decking *et al.*, 2020 ▸). LCLS-II, which is in commissioning, can operate up to 1 MHz (Galayda, 2018 ▸). The future FELs, such as LCLS-II-HE (Raubenheimer, 2018 ▸) and SHINE (Zhao, 2017 ▸), will also be able to operate up to 1 MHz. In these high-repetition-rate FELs, the time between two pulses can be as short as 1 µs, even down to sub-µs with the EuXFEL. The question is: do the optics have enough time to recover from the intense FEL pulse induced spikes in thermal deformation and elastic waves, so the subsequent FEL pulses will be always reflected by optics with acceptable performance? Is 1 µs too short – what is the minimum time between subsequent pulses? The answers to these questions are critical to define fundamental operating parameters such as repetition rate (1 MHz, 100 kHz, 10 kHz,…) versus pulse energy (10 µJ, 100 µJ, 1000 µJ,…). To address these questions, we are developing a finite-element model to simulate FEL pulse induced transient temperature and thermal deformation in X-ray optics.

Thermal deformation of X-ray optics for third-generation synchrotron light sources has been extensively studied by both experiment and simulation. For monochromator crystals, liquid nitro­gen (LN_2_) cooled silicon has been routinely used in third-generation light sources. Simulations using finite-element analysis (FEA) for the thermal deformation of cryo-cooled silicon crystals (Zhang, 1993 ▸; Zhang *et al.*, 2003 ▸; Tajiri *et al.*, 2001 ▸; Zhang *et al.*, 2013 ▸; Huang *et al.*, 2014 ▸; Brumund *et al.*, 2021 ▸; Khosroabadi *et al.*, 2022 ▸) have been performed for the steady state with average continuous power. The FEA simulations results are in excellent agreement with experimentally measured thermal deformation for slope error (Zhang *et al.*, 2013 ▸) and rocking curve broadening (Zhang *et al.*, 2003 ▸).

The repetition frequency of the hard X-ray FELs mentioned above varies from 50 Hz to greater than 1 MHz. The FEL power on the crystal is pulsed. The average power for low-repetition-rate FELs is less than 0.5 W and the thermal deformation of the crystal is not an issue. However, for MHz repetition rate FELs, the average power can be several hundreds of watts. This is comparable with the power load on the monochromator crystals in storage-ring-based light sources. Therefore, the thermal deformation of the crystal monochromator for high-repetition-rate FELs needs to be addressed. Initial FEA simulations of cryo-cooled diamond, natural silicon and isotopically enriched silicon crystals under pulsed FEL power loads were reported (Zhang, 2004 ▸) for the TESLA X-FEL proposal which later became the EuXFEL. That study addressed the 10 Hz transient effects of the pulse trains by assuming 4000 pulses spaced by 200 ns as a macro pulse train of 0.8 ms length. The temperature and thermal deformation do increase with time over the course of the pulse train. The pulse train is repeated every 100 ms. The time between pulse trains is long enough to allow the crystal to reach a uniform temperature: the maximum and minimum temperature tend to the same value but different than the initial temperature after 1 ms which is much shorter than the pulse train repetition time of 100 ms. Thermal deformation in slope error or height error, which depends on the temperature gradient but not on the uniform temperature level, drops to zero before the arrival of next pulse train after 100 ms. Thermal stress waves and vibrational analysis of a thin diamond crystal under high-repetition-rate FEL operation were investigated numerically (Yang *et al.*, 2018 ▸). That study discussed the high frequency of the thermal stress wave, later referred to as an elastic wave. The stability of Bragg reflectors in an X-ray free-electron laser oscillator (XFELO) has also been studied analytically and numerically (Bahns *et al.*, 2023 ▸) where temperature and strain distributions were discussed.

Up to now, however, we have not found more quantitative answers in the literature to the question of the recovery time of X-ray optics after the extreme peak-power FEL pulse induced spikes in thermal deformation, on the repetition rate for the required optics performance at a given pulse energy or the acceptable average power.

In this paper, we discuss time-domain modeling of the temperature and thermal deformation of a monochromator crystal under high-repetition-rate FEL pulses. The intrinsic thermal deformation of the crystal is created by the temperature gradient. The temperature response to the multiple FEL pulses is successive sharp peaks with a temperature increase during the arrival of the pulse, then followed by a decay with time until the next pulse arrives. This is the case in the area containing the beam footprint in the crystal. After enough pulses, the temperature at the period-end time of each pulse or just before the arrival of the next pulse reaches a constant value on a time scale which depends on the crystal size. That is what we call the ‘quasi steady-state’. The time to reach this state depends on the crystal geometry and material properties. The thermal deformation of the crystal at the period-end time determines the X-ray crystal optics quality since that is the state of the crystal exposed to the next XFEL pulse.

**Figure 1:**
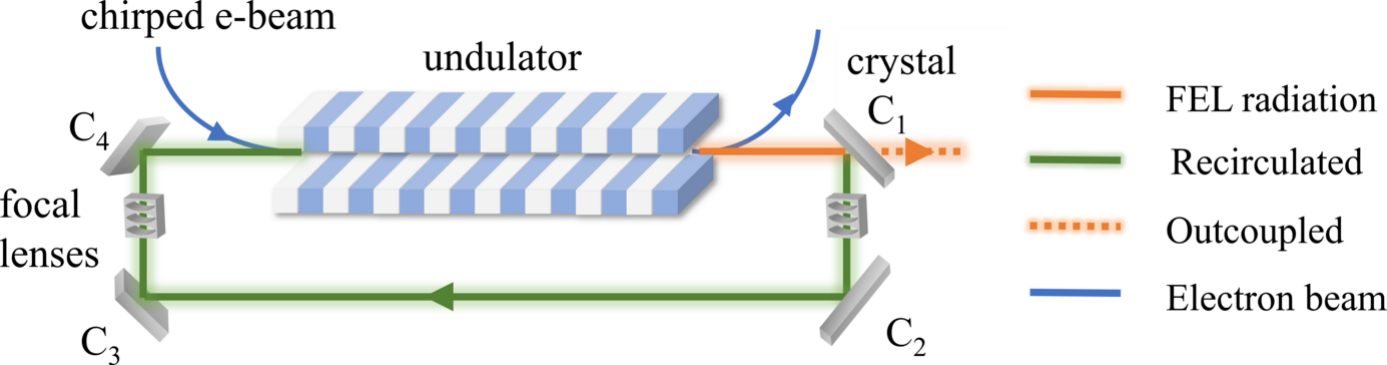
Schematic diagram of a CBXFEL with electron-beam-based Q-switching.

CBXFELs consist of an optical cavity in which the X-ray FEL beam is recirculated multiple times and interacts with the electron beam at the repetition rate, yielding a narrow bandwidth and high intensity (Huang & Ruth, 2006 ▸; Kim *et al.*, 2008 ▸; Marcus *et al.*, 2020 ▸; Rauer *et al.*, 2023 ▸; Margraf *et al.*, 2023 ▸). A schematic diagram of a CBXFEL is shown in Fig. 1 ▸ with an electron-beam-based Q-switching to outcouple the amplified FEL pulse. The X-ray FEL recirculating time is proportional to the optical path length in the cavity. For a 300 m-long cavity, the X-ray FEL recirculation time is 1 µs, or 1 MHz. The recirculation frequency must be an integer multiple of the electron beam pulse repetition frequency. The first crystal C_1_ outcouples the amplified FEL power and absorbs much more power than the other three crystals. At the first-turn time, this crystal in the cavity reflects the recirculating XFEL. Therefore, the thermal deformation of this crystal at the first-turn time needs to be investigated, in addition to the period-end time.

We note a collaboration between Argonne National Laboratory (ANL) and SLAC National Accelerator Laboratory (SLAC), supported by the US Department of Energy (DoE), to design and construct a proof-of-principle CBXFEL experiment at SLAC since 2019. The cavity is composed of four diamond crystals in Bragg geometry (Marcus *et al.*, 2020 ▸; Margraf *et al.*, 2023 ▸; Tang *et al.*, 2023 ▸).

Microsecond-scale, pulse-by-pulse transient simulation of the thermal deformation of X-ray optics has not been discussed in the literature. In this paper, we are focusing, in Sections 2–4, on the numerical simulation of the fundamental aspects of the transient effects of the crystal optics illuminated by FEL pulses. In Section 2 we describe the finite-element modeling and data processing of a water-cooled diamond crystal for a CBXFEL. We discuss the mesh size and transient analysis time increment and estimate the elastic wave propagation speed both from FEA and analytical analysis. In Section 3 we study the FEL power loading pulse length to determine an effective value for FEA without requiring the use of the actual fs-scale FEL pulse length, which would be too short for FEA modeling of a realistic crystal. In Section 4 we investigate the influence of the damping ratio on the attenuation of the elastic waves, on the numeric stability and convergence, and on the temperature gradient related thermal deformation. Finally, in Section 5 we present the pulse-by-pulse transient simulation of the temperature and thermal deformation of the crystal, the results concerning the influence of repetition frequency or repetition time and pulse energy on the thermal deformation, and in particular at the period-end time, and at the first-turn time in the cavity. We discuss the optics performance for wavefront preservation. Based on this analysis we draw clear conclusions about the transient effects of the thermal deformation, and recommend acceptable operational parameters, such as the upper bound of the pulse energy at any repetition frequency for wavefront preservation requirement.

## 2. Finite-element analysis

### 2.1. Diamond crystal for cavity-based FEL

The optical cavity in a CBXFEL is composed of four diamond crystals in Bragg geometry (Fig. 1 ▸). As an example of operating parameters, a photon energy of 9.831 keV corresponds to the diamond (400) Bragg reflection at 45.0°. To begin we consider the case of a 100 kHz electron beam repetition rate. For a 300 m-long round-trip cavity, the round-trip time is 1 µs. The narrow bandwidth XFEL pulse recirculates ten times as a seed in the cavity and then the chirped electron beam at 100 kHz repetition rate arrives in the undulator, is slightly compressed, or decompressed, resulting in a shift in the microbunching and the FEL wavelength. The majority of the FEL radiation spectrum is effectively switched out of the narrow reflection bandwidth of the diamond crystal for output. The remaining small portion of the spectrum within the reflection width is recirculated to seed the next electron bunch (Tang *et al.*, 2023 ▸). Therefore, this outcoupling crystal, noted as crystal C_1_ in the cavity, partially absorbs this output FEL power. We note that the power absorbed by the other three crystals, C_2_, C_3_ and C_4_, in the cavity is negligible compared with the power absorbed by the outcoupling crystal C_1_. The size of the diamond crystal C_1_ is assumed to be 5 mm × 5 mm square and 50 µm thick. At first, we assume a maximum pulse energy of 2.62 mJ at the 100 kHz repetition rate; this corresponds to an average FEL beam power of 262 W. The X-ray FEL beam size is 100 µm FWHM. The X-ray attenuation length of diamond at 9.831 keV is 1.226 mm; therefore, for 262 W input power, the power absorbed by a 50 µm-thick diamond crystal (400) oriented by 45° to the incident FEL beam is 14.7 W. In this paper, we focus on the thermal deformation of the first diamond crystal C_1_ since it absorbs much more power than the other three crystals.

### 2.2. Finite-element models (FEMs)

We assume that the first diamond crystal is water cooled. Some thermal and mechanical properties of diamond crystals are temperature dependent, such as thermal conductivity, thermal expansion and heat capacity. However, as the pulse-by-pulse transient FEA is computationally intensive, we do not want to introduce additional iterations in order to include temperature-dependent material properties. Furthermore, the use of constant room temperature material properties makes the simulation results (temperature raise, thermal deformation) scalable with FEL power and allows one to better understand the dependence of the thermal deformation on the power loading parameters, such as average power, pulse energy, repetition time and so on. When the temperature rise is not too high, for instance less than 100 K, this approximation leads to quite accurate numerical results. Thus, in general, all the results reported in this paper are qualitatively correct. In a future study we will use temperature-dependent material properties to compare the performance of water cooling and liquid nitro­gen cooling. The material properties of diamond used in this study are given in Table 1 ▸

**Figure 2:**
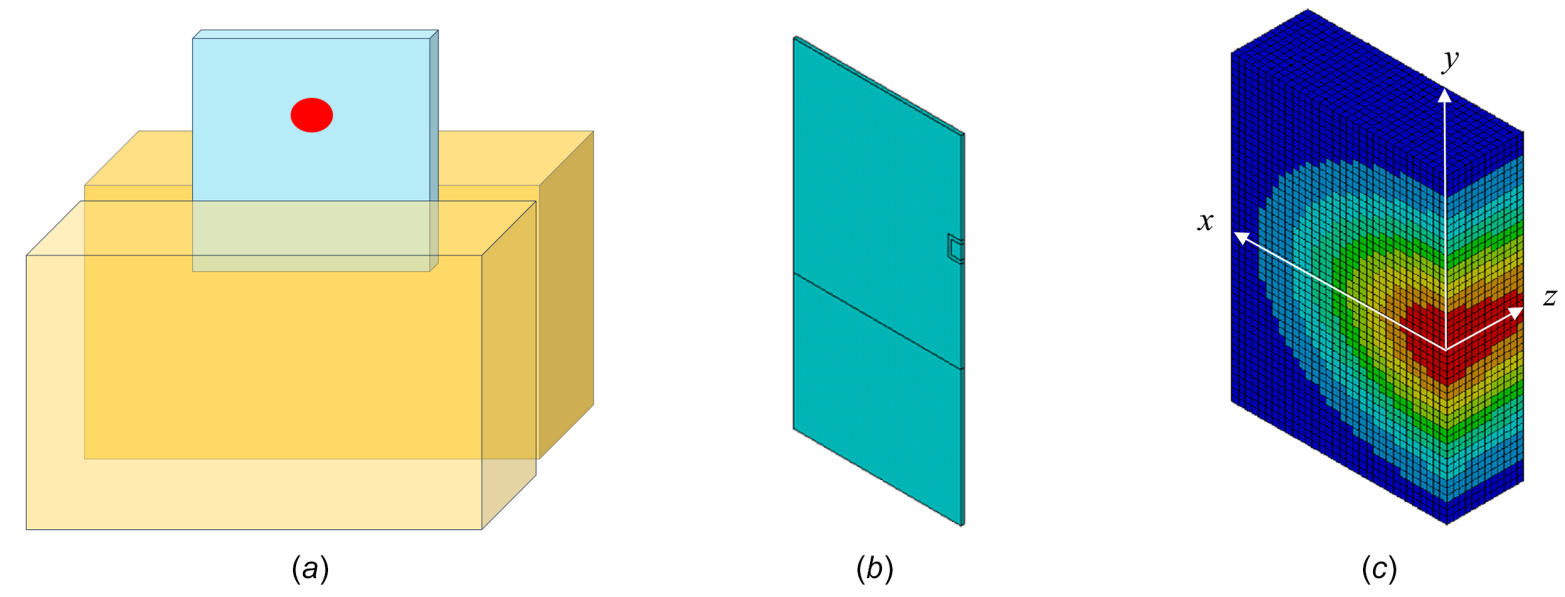
(*a*) Scheme of a diamond crystal (5 mm × 5 mm × 0.05 mm) held and cooled over 2 mm × 5 mm on both sides. (*b*) Finite-element geometrical model of a half diamond crystal, showing the symmetry condition on the right edge plane. (*c*) Zoom of the half volume of the crystal intercepted by the X-ray FEL with Gaussian power distribution in beam cross section (*x–y*) and in-depth (*z*) variable power absorption.

The diamond crystal is assumed to be held over 2 mm × 5 mm on both sides by copper cooling blocks as shown in Fig. 2 ▸(*a*). Indium foil can be applied between the diamond and copper interfaces to improve thermal contact, making the contact stress applied to the diamond crystal uniform, and increasing the structure damping of the diamond crystal and holder assembly. An effective cooling coefficient of *h*_cv-eff_ = 0.01 W mm^−2^ K^−1^ (Marion *et al.*, 2004 ▸; Khounsary *et al.*, 1997 ▸) is achievable in that case. Variable power load was applied to the diamond crystal in the following way: a Gaussian power distribution on the beam cross section (*x–y*), and exponentially varying power absorption along the beam propagation direction (*z*); see Fig. 2 ▸(*c*).

### 2.3. Temperature, thermal deformation, elastic waves in the crystal and data processing

In this study, we use the commercial finite-element analysis software *ANSYS* to compute the temperature and thermal deformation of the X-ray optics. In general, there are two approaches within *ANSYS* for this type of simulation: (1) sequential – firstly, perform the thermal analysis using a thermal element in the *ANSYS* library (for instance, SOLID90) to calculate the temperature distribution, then perform the mechanical analysis using a corresponding mechanical element with the temperature distribution as the loading condition to calculate deformation; (2) simultaneous – using a coupled field element (SOLID226) to calculate the temperature distribution and the thermal deformation simultaneously. When using the sequential approach for transient analysis, the mechanical transient analysis can be tricky, and the time increment needs to be carefully chosen consistent with the time increment used in the thermal analysis. For the FEM with a very large number of elements, the sequential method (1) can be up to 35% faster in computation time than the coupled method (2).

**Figure 3:**
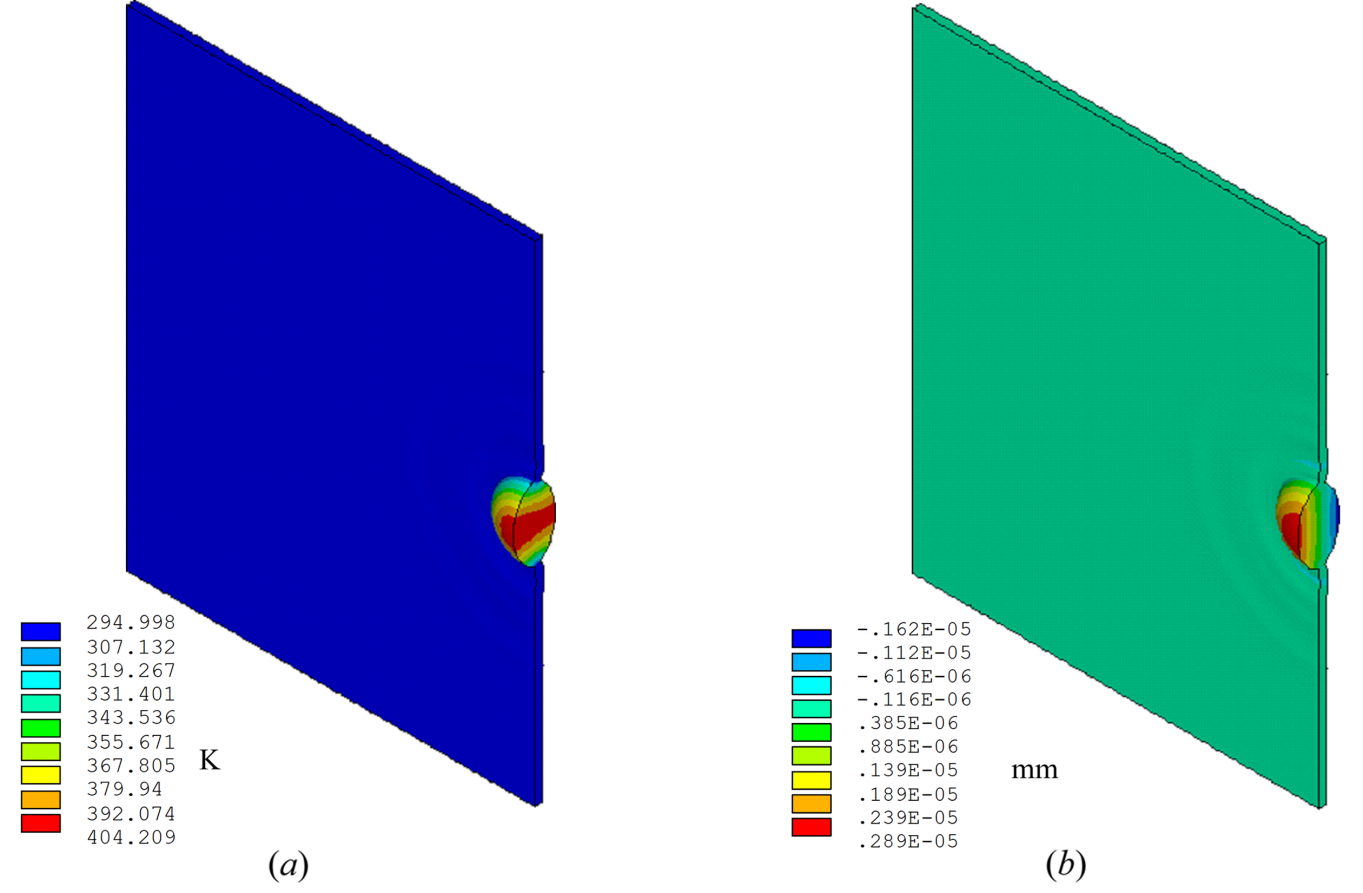
(*a*) Temperature and (*b*) displacement distribution in the crystal at 45 ns responses of the crystal under a 1 ns-long and 2.62 mJ of power loading pulse. Elastic waves can be observed on the displacement mapping (*b*). The thermal deformation scale is exaggerate here.

To accurately simulate high-frequency elastic waves, the coupled method (using coupled field element SOLID226) is better and more straightforward for full transient thermal deformation analysis. With the FEM described in Section 2.2, we can simulate the temperature and deformation responses of the diamond crystal under a certain number of FEL pulses over a few microseconds. We can obtain the temperature and deformation distribution in the crystal at any time, for example at 45 ns after the arrival of the first pulse as shown in Fig. 3 ▸, where the temperature and displacement mapping are only for half of the unfolded part of a 5 mm wide and 3 mm high crystal. Here we have temperature *T*(*x*,*y*,*z*,*t* = 45 ns) and displacement normal to the crystal surface *Uz*(*x*,*y*,*z*,*t* = 45 ns). In this simulation we assumed that the pulse power loading lasted 1 ns, with a pulse energy of 2.62 mJ. The power loading pulse length used here (1 ns) is much longer than the effective FEL pulse length; however, the simulated response of temperature and thermal deformation of the crystal after a few ns is independent of this power loading pulse length. This will be discussed further in Section 3. From the displacement distribution shown in Fig. 3(*b*) ▸, the elastic wave propagation can be observed.

In Section 5, we will see that the pulse energy used here (2.62 mJ) in the simulation is too high in terms of wavefront preservation for crystal optics at 100 kHz repetition frequency leading to a recommended pulse energy which is much less. Note again that the simulation results presented in this paper are scalable with FEL power (pulse energy, repetition frequency). Our focus, before discussing optics performance in Section 5, is on the fundamental aspects of the numerical simulation of the X-ray optics thermal deformation under pulse-by-pulse FEL.

**Figure 4:**
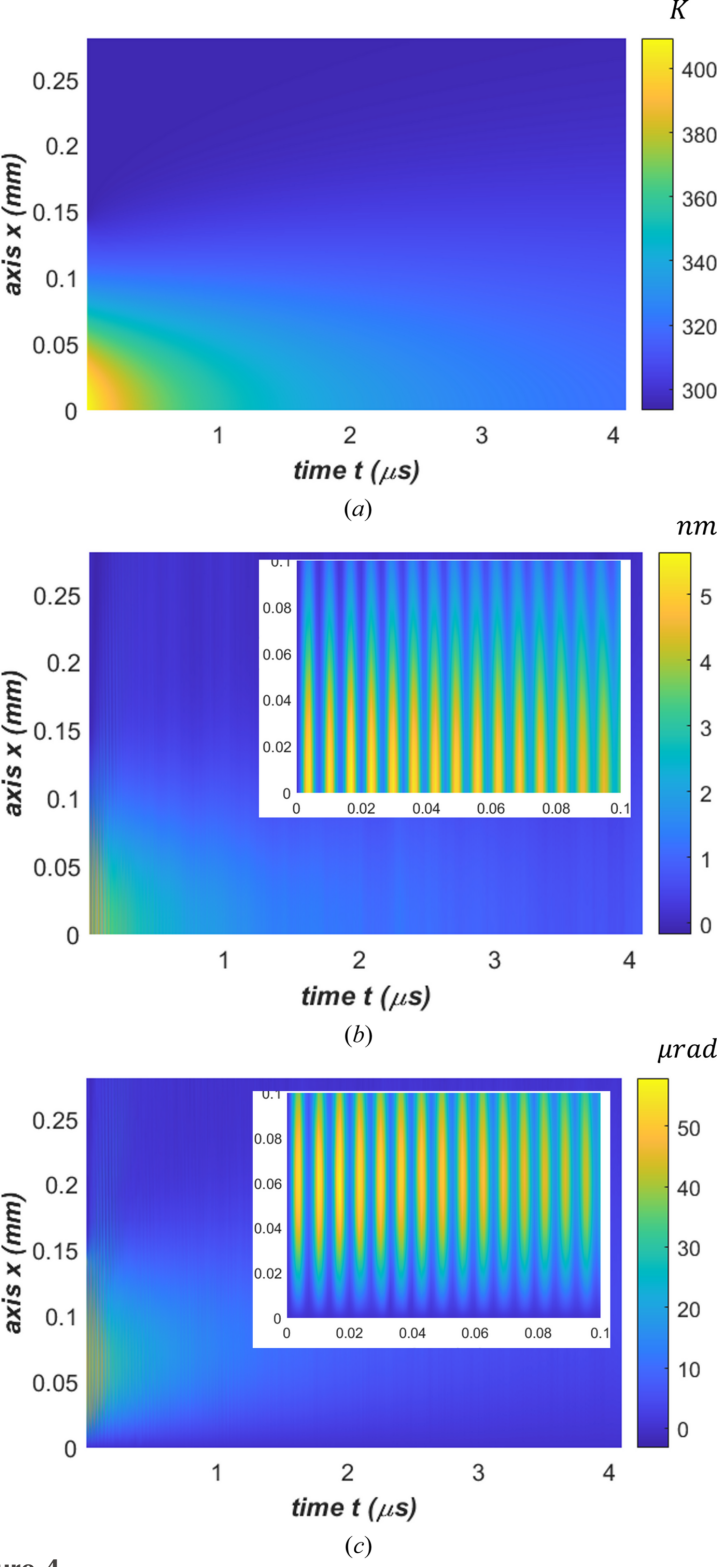
(*a*) Temperature, (*b*) displacement normal to the surface and (*c*) slope along the meridional axis (*x*) versus time, with a zoom on the upper right corner for both (*b*) and (*c*). Parameters used in FEA: power loading pulse length *t*_p_ = 1 ns, pulse energy *Q*_p_ = 2.62 mJ, time increment Δ*t* = 0.5, and default damping ratio ζ = 0.5%. Both temperature and displacement shortly reach the peak values at the center of the footprint *x* = 0, but the highest thermal slope error is located close to the edge of the footprint at *x* = FWHM/2 = 0.5 mm.

We are particularly interested in the thermal deformation along the center of the footprint on the crystal surface (*y* = 0, *z* = 0). The displacement along this line, *Uz*(*x*,*t*), can be used to calculate the thermally induced slope error at any time given by
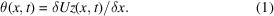
Temperature, displacement and slope error along this line (*y* = 0, *z* = 0) are shown in Fig. 4 ▸ versus time. The maximum temperature and maximum slope error reached shortly after the arrival of the FEL pulse are certainly too high above the required operating conditions for the crystal. For example, 50 µrad of slope error is greater than the Darwin width of the crystal. However, the FEL pulse duration is on the time scale of femtoseconds, but thermal deformation and temperature response are on the time scale of nanoseconds. The X-ray scattering of the first pulse by the crystal occurs well before the temperature and thermal deformation reached maximum and hence is unaffected. The diffracted beam quality of the next FEL pulse depends directly on the state of the crystal (temperature and thermal deformation) at the time just before its arrival, but not on the state with peak temperature and deformation immediately following that pulse. This is demonstrated by the fact that the crystal optics operate effectively with low-repetition-rate FELs with up to a few mJ pulse energy, in LCLS-I, SACLA, SwissFEL and PAL-XFEL. As mentioned by Zhang (2004 ▸), during the time between two FEL pulses, the crystal is recovering towards its initial state, and temperature and thermal deformation in the crystal decrease with time until the arrival of next FEL pulse. This is the so-called crystal optics recovery effect. For a lower repetition rate, and hence a longer time between two pulses, the more complete the recovery effect. These recovery effects are analyzed in detail in Section 5.2. Furthermore, as mentioned in Section 2.1, simulation results (temperature rise, thermal deformation) are scalable with FEL power or pulse energy. This allows us to determine the acceptable parameters, such as pulse energy versus repetition frequency, from simulation results with any pulse energy.

The parameters used in the FEA are listed in the caption of Fig. 4 ▸. All the results shown in Fig. 4 ▸ reach peak values shortly after the arrival of the pulse. As expected, the peak temperature and displacement are located at the center of the footprint *x* = 0, but the peak slope is found close to the edge of the footprint at *x* = FWHM/2 = 0.5 mm. An elastic wave can be observed in the vertical displacement, *Uz*(*x*,*t*), in Fig. 4 ▸(*b*) (zoom), and in the slope error, θ(*x*,*t*), in Fig. 4 ▸(*c*) (zoom). Fig. 4 ▸(*b*) shows the displacement normal to the crystal surface (*z*-axis) along the *x*-axis [see Fig. 2 ▸(*c*) for a definition of the coordinate system] versus time. The elastic wave manifests in a way such that the thin diamond crystal surface moves up and down in the *z*-axis direction and can be described as a vibrational displacement. The results in Fig. 4 ▸(*b*) show two components of displacement *Uz*(*x*,*t*): (1) vibrational and (2) temperature gradient related. On the top surface of the crystal the total displacement is always positive and therefore the elastic wave related displacement is smaller than the temperature gradient induced displacement. The elastic wave related fluctuation is shown by the vertical stripes in both Figs. 4 ▸(*b*) and 4 ▸(*c*). There are no vertical stripes in Fig. 4 ▸(*a*) for temperature. The frequency of the elastic wave will be discussed in detail in Section 2.5. The vibrational component of the displacement has a fine time structure of about 6 ns and is attenuated over time by damping in the diamond crystal. The temperature gradient related component of displacement decreases with time as temperature becomes uniform across the entire beam footprint area of the crystal on a time scale of greater than a few microseconds. We can also calculate the standard deviation of the height error and slope error over the beam footprint on the crystal from the results in *Uz*(*x*,*t*) and θ(*x*,*t*).

For a given point in the crystal, we can extract the displacement or slope error response in time, then apply a fast Fourier transform (FFT) to calculate the power spectral density (PSD). The elastic wave propagation frequency can be defined from the peaks in the PSD.

To verify that the results in temperature, displacement and slope error of the crystal are reliable and accurate, we investigate below the choice of the fundamental parameters such as mesh size, time increment, pulse length and damping ratio to be used in our FEA, and their influences.

### 2.4. Mesh size and time increment

There are several time scales in the transient thermal deformation of the X-ray optics under a high-repetition-rate FEL beam: (1) the FEL pulse length (*t*_p_) which is in the range 10–500 fs, (2) the repetition time (*t*_per_) which is ≥1 µs, and (3) the time to reach quasi steady-state in temperature and in thermal deformation which can be on the scale of ms, depending on the size and material of the optic. Qualitatively, every one of the fs-scale FEL pulses induces a spike in temperature and thermal deformation and drives elastic waves in X-ray optics. Between pulses, the temperature tends to stabilize, and the thermal deformation and elastic waves decrease to a quasi steady-state level. Clearly this recovery will be more complete when the repetition time is longer. Both temperature and thermal deformation should tend to the quasi steady-state level with the accumulation of a large number of pulses.

**Figure 5:**
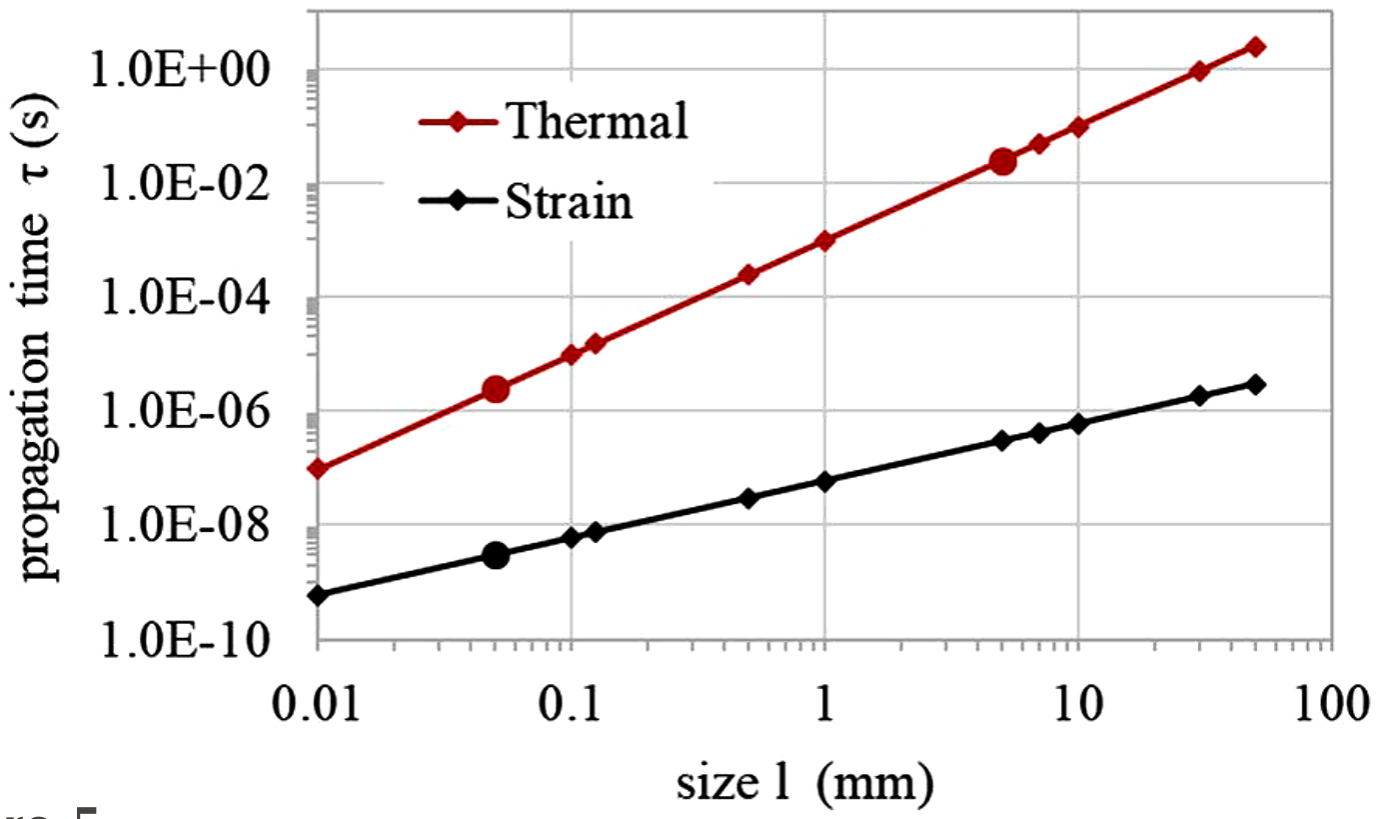
Thermal diffusion and strain propagation time constants for diamond crystal at different typical sizes.

For transient thermal deformation and elastic waves, there are two different time constants for thermal diffusion and strain (or displacement) propagation. For a typical length *l* of an optic, the thermal diffusion time τ_th_ is of the order of magnitude defined by
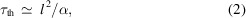
where α is the thermal diffusivity of the material. The strain propagation time τ_d_ is of order defined by
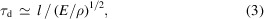
where *E* and ρ are Young’s modulus and the density of the material, respectively. Thermal diffusivity α can be calculated from density ρ, heat capacity *C*_p_ and thermal conductivity *k* as α = *k*/ρ*C*_p_. For a diamond crystal, thermal diffusion and strain propagation times versus size *l* are shown in Fig. 5 ▸ based on the material properties given in Table 1 ▸. From the results in Fig. 5 ▸ we observe that (i) the strain propagation time is much shorter than the thermal diffusion time, or the strain propagation length is much longer than the thermal diffusion length with the same time interval; and (ii) denoting the slope of the strain propagation time versus size *l* as *k*_τd_, then the elastic wave propagation speed can be estimated as 1/*k*_τd_ to be 17 km s^−1^. For a 50 µm-thick and 5 mm square crystal, the thermal diffusion and strain propagation times are, respectively, about 3.2 µs and 32 ms. Under a short pulse power loading on a diamond crystal of typical size *l*, the time to reach steady-state *t*_ss_ can be estimated by exp(−*t*_ss_/τ_th_) << 1; for instance, exp(−*t*_ss_/τ_th_) = 0.05, or *t*_ss_ = 3τ_th_.

**Figure 6:**
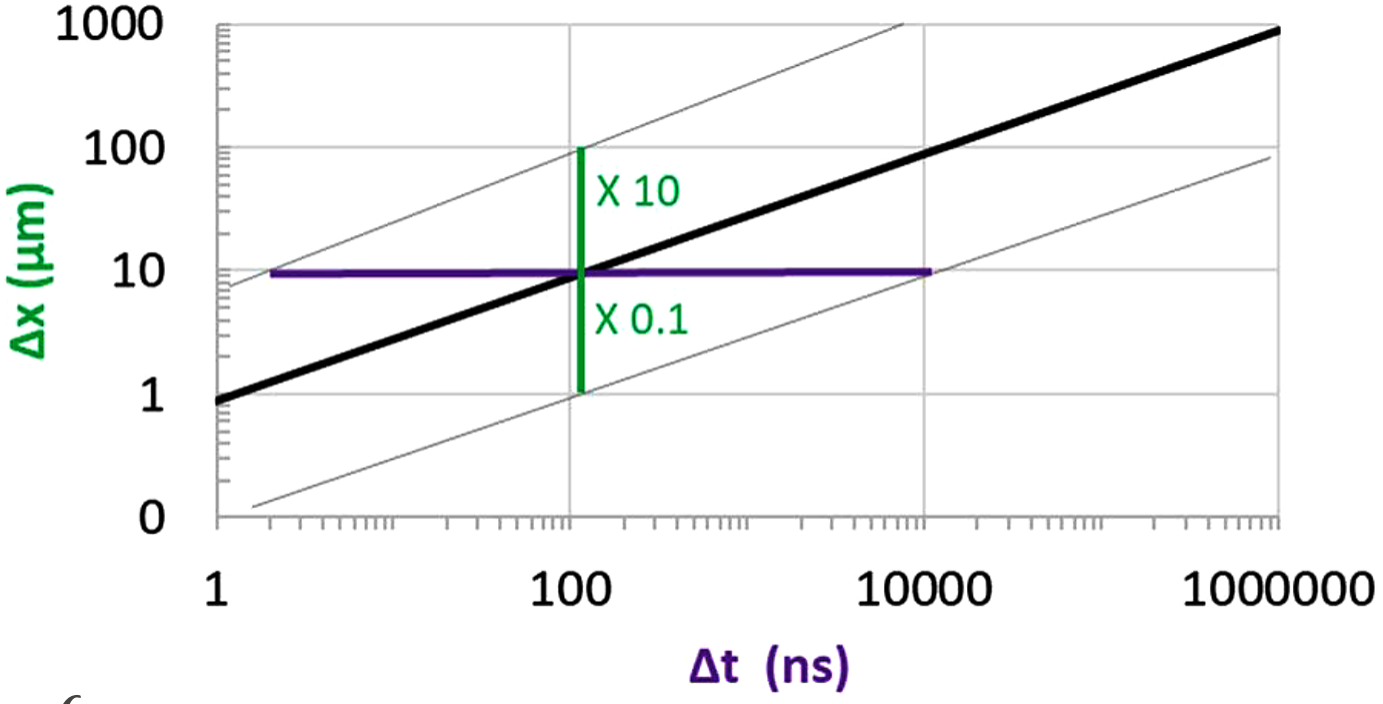
Time increment and mesh size relation for transient FEA of a diamond crystal.

Since the FEL pulse length is sub-ps, the time increment in transient analysis of the thermal deformation of the X-ray optics could be down to the sub-ps level. On the other hand, when using FEA to simulate transient thermal deformation and elastic waves, the time increment Δ*t* and mesh size Δ*x* should satisfy certain conditions to ensure numerical convergence, accuracy and a reasonable FEA model size and computation time. In other words, for a given time increment Δ*t*, the mesh size Δ*x* should be small enough to have the required simulation accuracy and but not too small in order to avoid too large a number of elements. Over this time increment, the strain propagation length is several orders of magnitude greater than Δ*x* so the mesh size appears very small and thus the numerical accuracy should be very good for strain propagation. Therefore, we can focus on thermal diffusion to define the relation between the time increment Δ*t* and mesh size Δ*x*. Within the time increment Δ*t*, heat propagation over Δ*x* equal to 10% of thermal diffusion length can be considered as a mesh size Δ*x* that would be small enough. Similarly, heat propagation over Δ*x* equal to ten times the thermal diffusion length can be considered as the mesh size Δ*x* being large enough. Fig. 6 ▸ shows the range of the mesh size for any given time increment for a diamond crystal.

For a diamond crystal of size 5 mm × 5 mm × 0.05 mm, and a strain propagation speed of about 17 km s^−1^, the time for strain traveling the crystal thickness is about 3 ns. Therefore, a time increment Δ*t* of about 1 ns is necessary for accurate simulation of the elastic wave propagation. The mesh size Δ*x* should be smaller than 10 µm, which leads to ∼10^6^ regular meshing elements for the diamond crystal. Such an FEA model size can be run within a few days to simulate the transient response of a few microseconds on a Windows workstation. For a larger crystal size, for instance a bulk silicon crystal of 50 mm × 50 mm × 30 mm, the typical size of the silicon monochromator crystal for X-rays, a time increment Δ*t* of about 1 µs is necessary for accurate simulation of the elastic wave propagation. The mesh size Δ*x* should be smaller than 100 µm leading to about 10^8^ elements and too large of an FEA model for practicable transient analysis on a Windows workstation.

**Figure 7:**
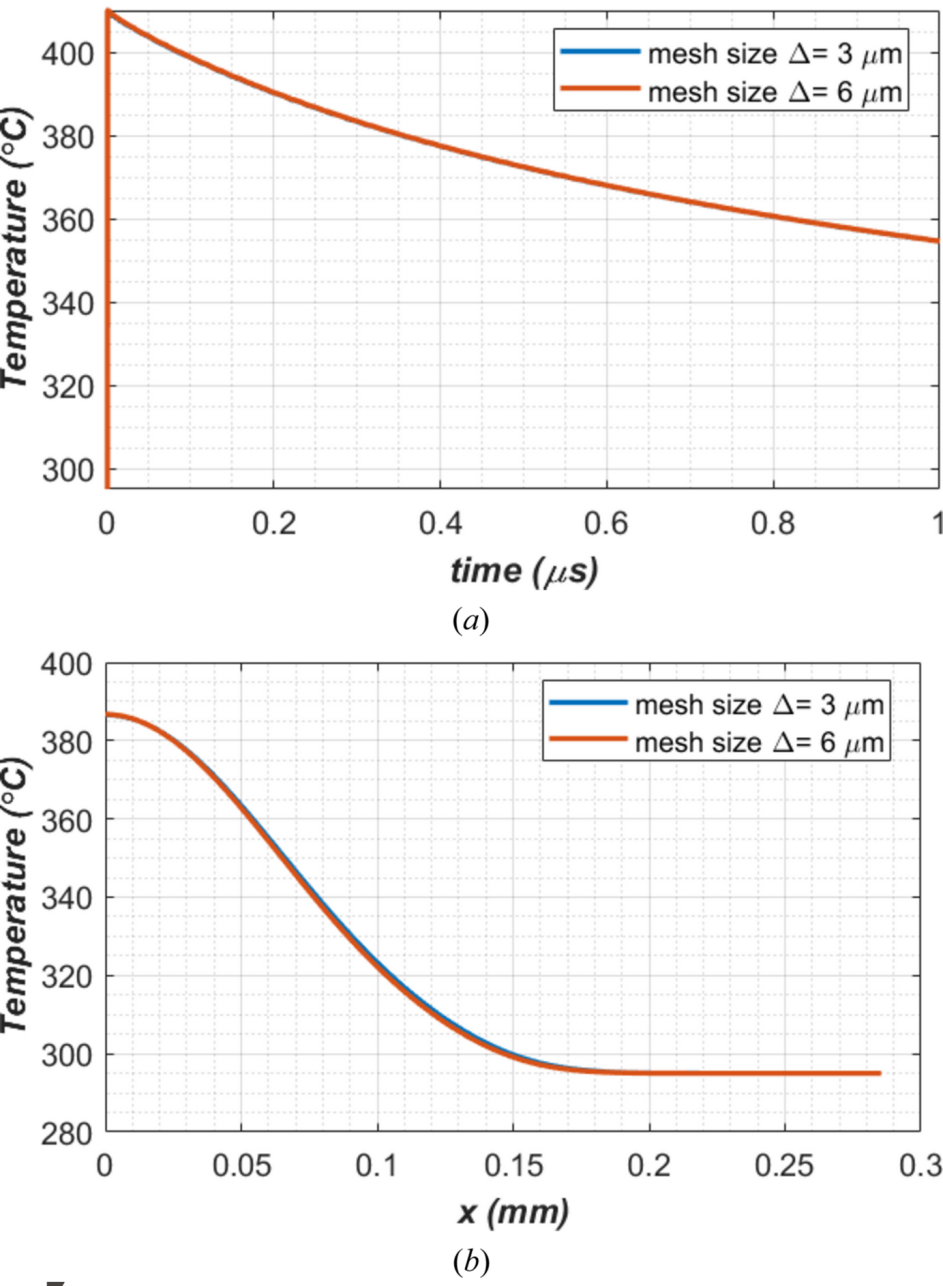
Comparison of simulation results with the smallest element size in the beam footprint on the crystal between 3 and 6 µm. (*a*) Temperature at the center of the beam footprint versus time. (*b*) Temperature profile along the center of beam footprint axis (*x*) at 0.25 µs after arrival of the FEL pulse.

We have performed full transient thermal deformation analyses with a time increment Δ*t* = 1 ns and the smallest mesh size of either 3 or 6 µm. To compare these two cases, we show the temperature at the center of the beam footprint versus time in Fig. 7 ▸(*a*), and the temperature along the *x*-axis at *t* = 0.25 µs after the arrival of the FEL pulse in Fig. 7 ▸(*b*). There are no visible differences between the results of these two cases which suggests that the mesh size is reasonable. Therefore, in this study, we will mostly use a mesh size of 6 µm.

### 2.5. Elastic wave propagation speed and frequency

**Figure 8:**
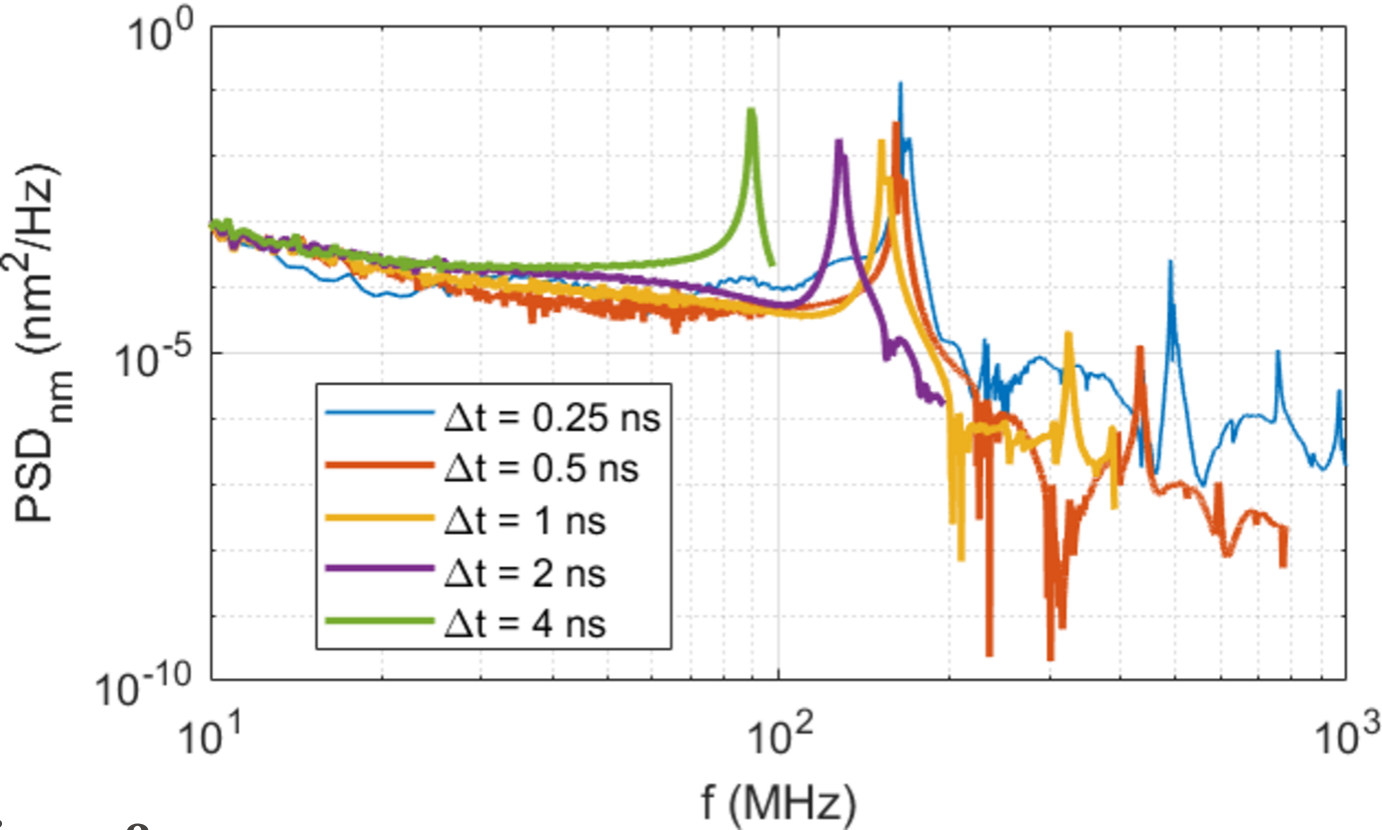
PSD of the displacement at the center for the beam footprint on the crystal calculated with different time increments Δ*t* = 0.25, 0.5, 1, 2 and 4 ns used in FEA. The pulse energy and the power loading pulse length are, respectively, *Q*_p_ = 2.62 mJ and *t*_p_ = 1 ns.

Accurate numerical results for elastic wave propagation speed and frequency need sufficiently small time increments for the transient simulation. To investigate the influence of the time increment Δ*t* on the simulation results, we have carried out FEA simulations over 4 µs with Δ*t* = 0.25, 0.5, 1, 2, 4 ns uniform time increments after full pulse power loading. The pulse energy and the power loading pulse length were, respectively, set for *Q*_p_ = 2.62 mJ, *t*_p_ = 1 ns with a time increment of *t*_p_/10 during the power pulse loading. From the FEA results, we have calculated the PSD of the displacement at the center of the beam footprint on the crystal surface. Results of the PSD of *Uz*_c_(*t*), the displacement normal to the crystal surface at the center of the footprint, are plotted in Fig. 8 ▸. The sampling time dts = Δ*t* is related to the bandwidth of the PSD as *f*_max_ = 1/Δ*t*/*N*_nyq_, where *f*_max_ is the maximum frequency of the PSD in the bandwidth, and *N*_nyq_ = 2.56 is the Nyquist factor. It is clear that a smaller time increment yields a larger PSD bandwidth, and longer simulation time. The frequency resolution depends on the length of the time window. Transient simulation with one pulse was performed over 4.096 µs, which was chosen as the window length for the FFT. The frequency resolution is then 0.244 MHz. The first peak between 80 and 200 MHz on each PSD curve is related to the elastic wave in terms of the displacement *Uz*_c_. The frequency corresponding to this first peak is called the fundamental frequency *f*_1_ of the elastic wave and varies with the time increment Δ*t* used in the FEA.

**Figure 9:**
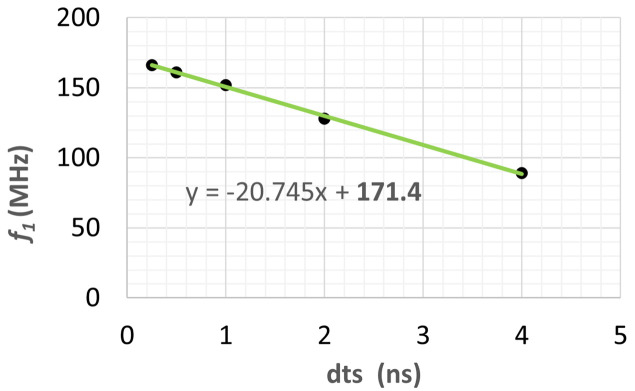
Fundamental frequency *f*_1_ of the elastic wave calculated with different sampling time (time increment Δ*t*) from the PSDs shown in Fig. 8 ▸.

Fig. 9 ▸ shows that the value of this fundamental frequency *f*_1_ is a linear function of the time increment Δ*t* used in the FEA. The smaller the time increment Δ*t* in the FEA, the more accurate the transient simulation results, especially for high frequency elastic waves. When the sampling time dts or time increment Δ*t* tend to 0, the value of the fundamental frequency tends to *f*_1_ = 171.4 MHz. This value is in good agreement with the longitudinal wave propagation frequency calculated using the following analytical formula,
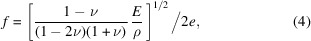
where *e* is the thickness of the diamond crystal mentioned in Section 2.1 (*e* = 50 µm), and the of material properties (*E*, ν, ρ) values are given in Table 1 ▸. The analytical result is *f* = 170.5 MHz. In equation (4), the elastic wave propagation speed *v*_e_ is used, which is
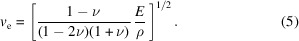
The corresponding elastic wave propagation speed in diamond is then *v*_e_ = 17.0 km s^−1^, independent of the geometry of the diamond crystal.

## 3. Power loading pulse length

The process of the interaction of a short FEL pulse (∼10–500 fs) with matter is quite complex (London *et al.*, 2001 ▸). The X-rays are first absorbed mainly by inner-shell photoionization, then followed by photoelectron emission, photoionization, collisional ionization and free-electron slowing down. The electrons will thermalize with the ions and neutral atoms in the material and then finally recombine with the ions. The time scale of this thermalization and electron–ion recombination is on the ps time scale. Thermal waves and non-equilibrium heat diffusion after the FEL pulse on time scales of fs and ps (Wang *et al.*, 2011 ▸) are not our concern since we are interested in the crystal state just before the arrival of the subsequent pulse, which is longer than the 1 µs time scale. Therefore, we can simplify the FEL pulse power loading on the X-ray optics as a longer pulse, for instance a 1 ns pulse for the convenience of numerical simulation. This ns scale power loading time is in the range of the equilibrium heat diffusion in the heat transfer process and therefore the Fourier heat transfer law is valid (Wang *et al.*, 2011 ▸) and it is thus appropriate to use commercial FEA software to simulate transient temperature and thermal deformation of the crystals under power loading pulses of ns length.

**Figure 10:**
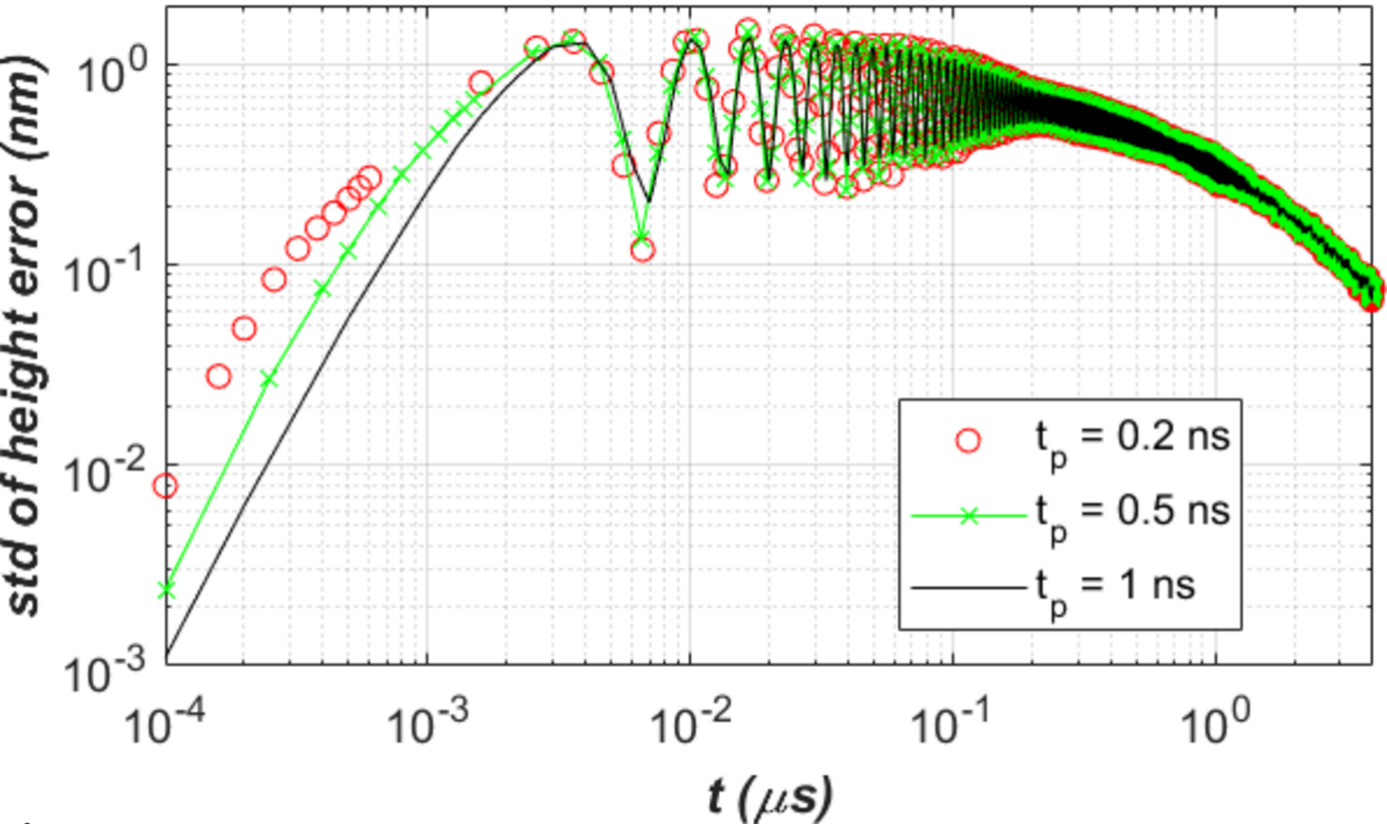
Standard deviation of the height error over the beam footprint on the diamond crystal surface: comparison between results with power loading pulse length *t*_p_ = 0.2, 0.5 and 1 ns. Results in height error converged in less than 10 ns after power loading.

A shorter power loading pulse needs smaller time increments or a larger number of time steps to reach the response of the optics up to a few or tens of microseconds. To investigate the relevance of this approximation, we have performed simulations with power loading pulse lengths *t*_p_ = 0.2, 0.5 and 1 ns. Fig. 10 ▸ shows the standard deviation of the height error over the beam footprint on the diamond crystal surface for three different power loading pulse lengths. Results in height error converge in less than 10 ns after the power loading. Therefore, we can use a 1 ns pulse length for the power loading and correctly predict the thermal deformation responses of the diamond crystal at the FEL repetition time scale (≥1 µs for LCLS-II and LCLS-II-HE).

## 4. Damping of elastic waves

**Figure 11:**
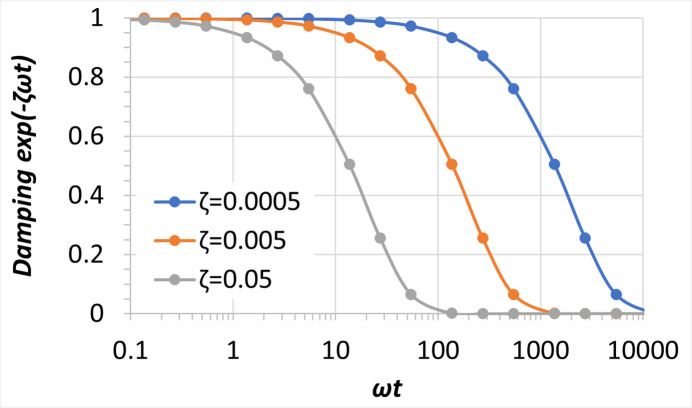
Attenuation factor versus ωt for different damping ratio ζ.

The oscillation of thermal deformation including elastic waves in the diamond crystal after a FEL pulse is attenuated with time following an exponential law: exp(−ζω*t*), where ζ and ω are the damping ratio of the diamond crystal and angular frequency of the oscillation, respectively. This vibration attenuation term is plotted in Fig. 11 ▸ for ζ = 0.0005, 0.005 and 0.05 versus ω*t*. We can define a significant attenuation as ζω*t* = 5; this corresponds to an attenuation factor of 0.0067. For the case described in Section 2.1 (50 µm-thick diamond crystal), the fundamental elastic wave propagation frequency is *f*_1_ = 171.4 MHz, or ω_1_ = 27.3 MHz. The times to reach a significant attenuation of all the elastic waves are then 367, 36.7 and 3.67 µs for ζ = 0.0005, 0.005 and 0.05 respectively.

**Figure 12:**
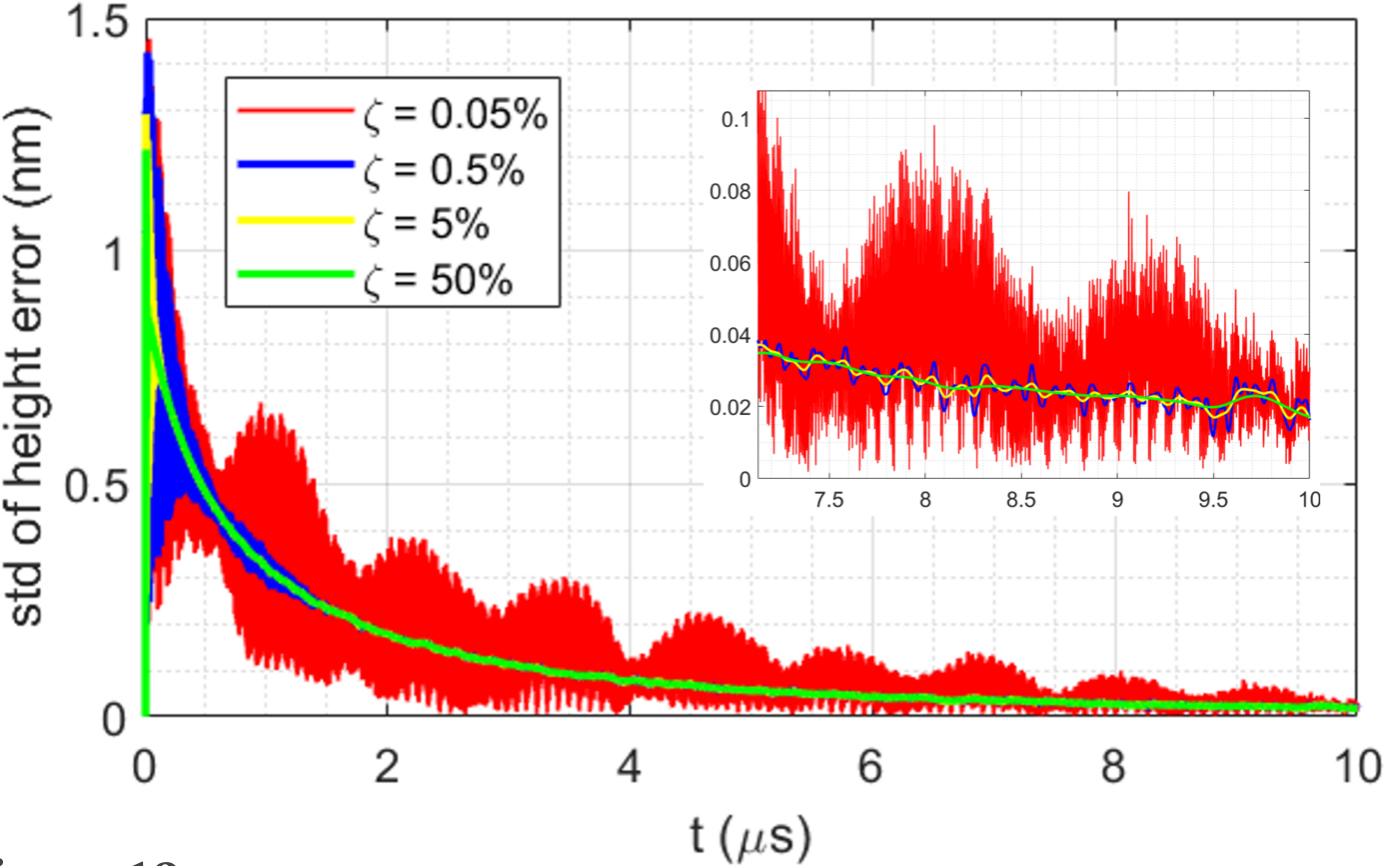
Thermal deformation in standard deviation of height error on the center of the beam footprint over 2 × FWHM versus time, with different damping ratio of ζ = 0.0005, 0.005, 0.05 and 0.5.

Full transient FEA simulations have been carried out for a 50 µm-thick diamond crystal, time increment Δ*t* = 2 ns, 2.62 mJ of pulse energy and 1 ns pulse length, response over 10 µs, and damping ratio ζ = 0.0005, 0.005, 0.05 and 0.5. Results in standard deviation of the height error over the beam footprint on the diamond crystal are depicted in Fig. 12 ▸.

After each pulse and temperature spike in the crystal, the temperature distribution tends to become uniform, that is, the temperature gradient is decreasing with time. The thermal deformation is directly related to the temperature gradient. Therefore, the thermal deformation or the slope error, in general, decreases with time as well as the amplitude of elastic waves. In the time response of the diamond crystal to the 2.62 mJ pulse, the elastic wave propagation can be limited to only the first few µs if the damping ratio is greater than 0.005. The elastic waves can be dominant in the thermal deformation if the damping ratio is less than 0.0005. These results are consistent with the discussion above and the analytical data shown in Fig. 11 ▸.

Material damping properties in nanomechanical resonators have been discussed by several authors (Tao *et al.*, 2014 ▸; Hutchinson *et al.*, 2004 ▸; Najar *et al.*, 2014 ▸; Denu * et al.*, 2017 ▸). The quality factor from those studies ranges between 4000 and 150000. This corresponds to the largest damping ratio of 0.00012. Since the thin diamond crystal for a CBXFEL would be held by cooling blocks with indium foil layers between the diamond crystal and the cooling blocks, these interfaces should significantly increase the damping in the diamond crystal to ζ ≃ 0.01, and possibly larger.

We note that further experimental studies are necessary to investigate the effective damping in the diamond crystal. Crystal size, especially the thickness for the CBXFEL application, and the environment around the crystal–boundary conditions will influence the elastic wave damping by changing both the frequency of the elastic waves and the damping ratio. By increasing the period time or decreasing the repetition rate, one should be able to reach a state where the elastic waves are attenuated to a negligible level.

In the following sections, we will assume that elastic waves can be totally attenuated after 10 µs, the 100 kHz repetition rate. The fundamental question is what the residual thermal deformation of the crystal is when the next FEL pulse hits the crystal. In the example shown in Fig. 12 ▸, for instance, at 10 µs, the residual slope error is not zero. This can influence the diffraction of the next pulse if the repetition rate is 100 kHz. So far, we have described the thermal deformation of the crystal from the first pulse. How the residual thermal deformation builds up when accumulating pulses is the key to addressing the fundamental question mentioned previously.

## 5. Pulse-by-pulse transient thermal deformation

To simulate the pulse-by-pulse transient thermal deformation of the crystal under a high-repetition-rate FEL, we have, in the previous section, discussed the fundamental parameters to be used in the FEA. These include mesh size, time increment, power loading pulse length and damping ratio. In this section we focus on the full transient simulation and the results on thermal deformation. First, we need to know the time to reach quasi steady-state, where the temperature and thermal deformation still vary with time, but the variation pattern is repeated at the FEL pulse repetition frequency. Then we simulate the thermal deformation at different FEL repetition rates, discuss the influence of the repetition times (*t*_per_) or repetition frequency (*f*_rep_) and define operational parameters such as pulse energy versus repetition frequency for wavefront preservation of crystal optics.

### 5.1. Time to reach quasi steady-state

For the diamond crystal described in Section 2, we have performed steady-state and transient simulations assuming a constant (continuous wave – CW) power loading of 262 W. Transient analyses have been performed using two different values of the damping ratio, ζ = 0.005 and 0.5. The first value is the *ANSYS* default value when using a coupled field element (SOLID226). From the discussion in Section 2.4, the steady-state of the 5 mm × 5 mm × 0.05 mm diamond crystal is reached in about 50 ms. As mentioned in Section 4, we are focusing on the temperature gradient related thermal deformation, but no longer on FEL pulse induced elastic waves, therefore we can use automatic variable time increments in the transient analysis. This permits a decrease in the number of time steps and pushes the transient simulation under CW power loading to 100 ms to a reasonable computation time, less than 5 h, on a Windows workstation (Intel Xeon CPU E5-2687W v4 @ 3.00 GHz, 12 cores, 160 GB RAM, 8 TB HD).

**Figure 13:**
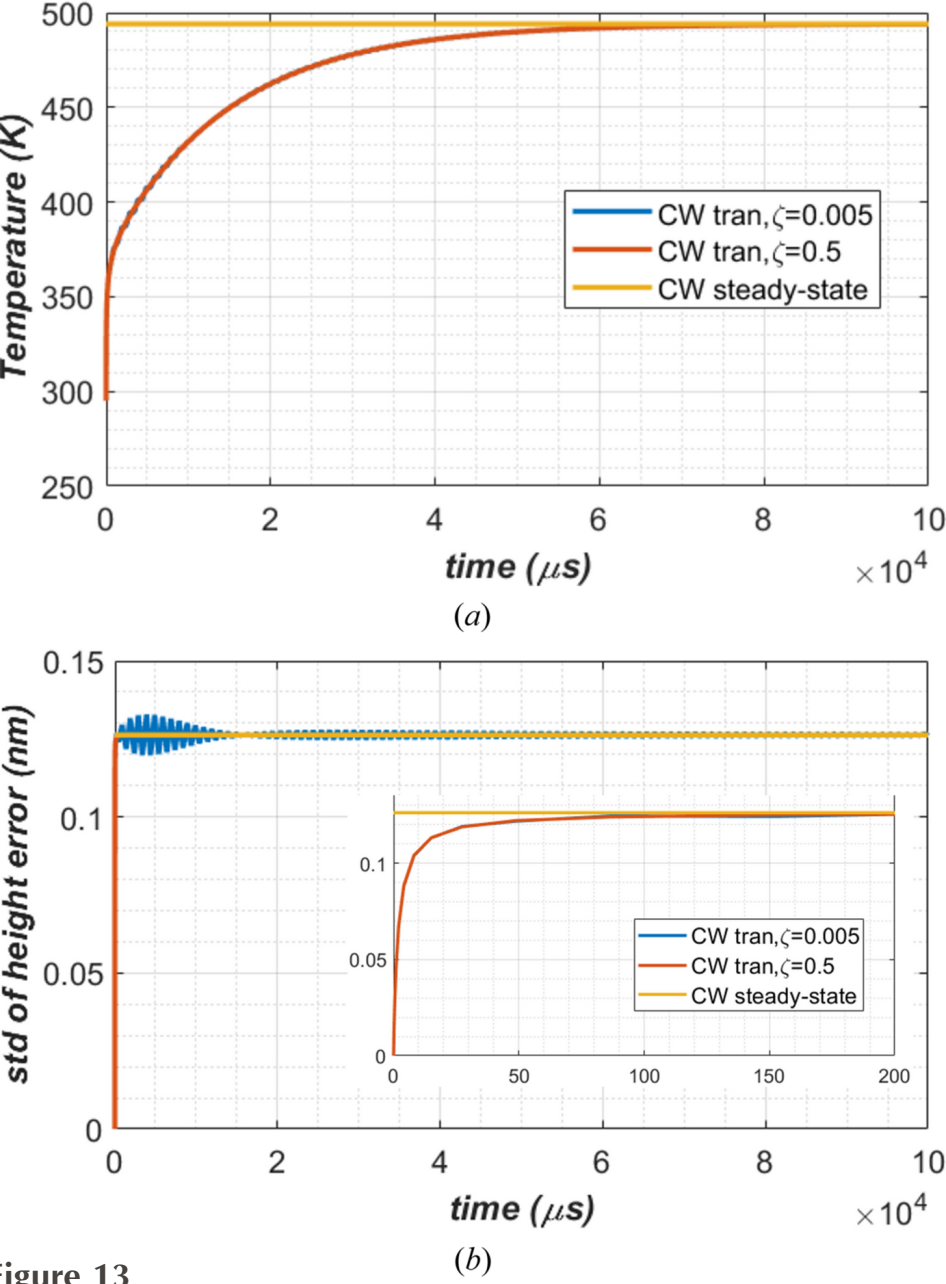
(*a*) Temperature at the center of the footprint, (*b*) thermal deformation in standard deviation of height error over the beam footprint versus time and zoom over 200 µs. Heat load is a constant power of 262 W. Three cases are compared: CW steady-state, and CW transient with damping ratios of ζ = 0.005 and ζ = 0.5.

Results for the temperature at the center of the footprint (*T*_0_) and the standard deviation of height error over the beam footprint on the diamond crystal surface are shown in Fig. 13 ▸. From these results we have following observations:

(1) The thermal deformation under CW power loading from transient analysis converges to those from steady-state analysis, independent of the damping ratio ζ = 0.005 or 0.5. This implies that the value of the damping ratio has no influence on the very low frequency thermal deformation which is the case for temperature gradient related thermal deformation. However, the damping ratio does have a significant influence on the high frequency elastic waves as presented in Section 4, and on numerical stability as well.

(2) When time steps are significantly longer than 2 ns, the default damping ratio ζ = 0.005 is not enough to avoid numerical instability. However, a large damping ratio ζ = 0.5 leads to smooth transient analysis results.

(3) The temperature reaches steady-state in about 50 ms, consistent with the analytical estimation discussed in Section 2.4.

(4) The time to reach steady-state for thermal deformation is about 50 µs. As the thermal deformation is created by the temperature gradient, especially in the direction of the thickness of the diamond crystal, this means that the temperature gradient reaches steady-state faster than the temperature itself. The 3D heat conduction under the footprint in the crystal increases the characteristic length *l*_cond_ to greater than the crystal thickness. Assuming a factor of three increase due to the 3D heat conduction, *l*_cond_ is about 0.05 × 3 = 0.15 mm. This leads to a thermal diffusion time of 86 µs for the temperature gradient, or thermal deformation. This number is consistent with the FEA results shown in Fig. 13 ▸(*b*).

### 5.2. Pulse-by-pulse transient responses versus repetition frequency

To address the question of the recovery time of optics under high-power FEL pulse induced spikes in thermal deformation, we have studied a few cases of power loading with the same average power *P*_av_ = 262 W but different repetition times *t*_per_ = 1, 2, 5, 10, 20 and 50 µs. The corresponding pulse energies are *Q*_p_ = 0.262, 0.524, 1.31, 2.62, 5.24 and 13.1 mJ, respectively. The relation between average power, pulse energy and repetition time is given by *Pv* = *Qp*/*t*_per_. The repetition rate is related to repetition time as *f*_rep_ = 1/*t*_per_.

Full transient thermal deformation simulations of the diamond crystal have been performed from the first pulse to the *N*th pulse up to 200 µs – the time to reach quasi steady-state for this diamond crystal. Here *N* is equal to 200/*t*_per_ = 200, 100,…, 10, 4. As a reference, the transient simulation with CW power of 262 W was also performed over 200 µs. The computation time with our Windows workstation lasts up to two weeks (for the case of *t*_per_ = 1 µs and 200 pulses). It should be noted that continuous two weeks of computation can be challenging due to possible power outages and other IT interruptions. For example, for the case of *t*_per_ = 1 µs and 200 pulses, the computation was interrupted by an IT incident after more than 11 days; only the responses of 169 pulses were calculated. To mitigate these problems, a cluster or a supercomputer could be used for this simulation but this requires an appropriate *ANSYS* license (a large number of cores can be used).

**Figure 14:**
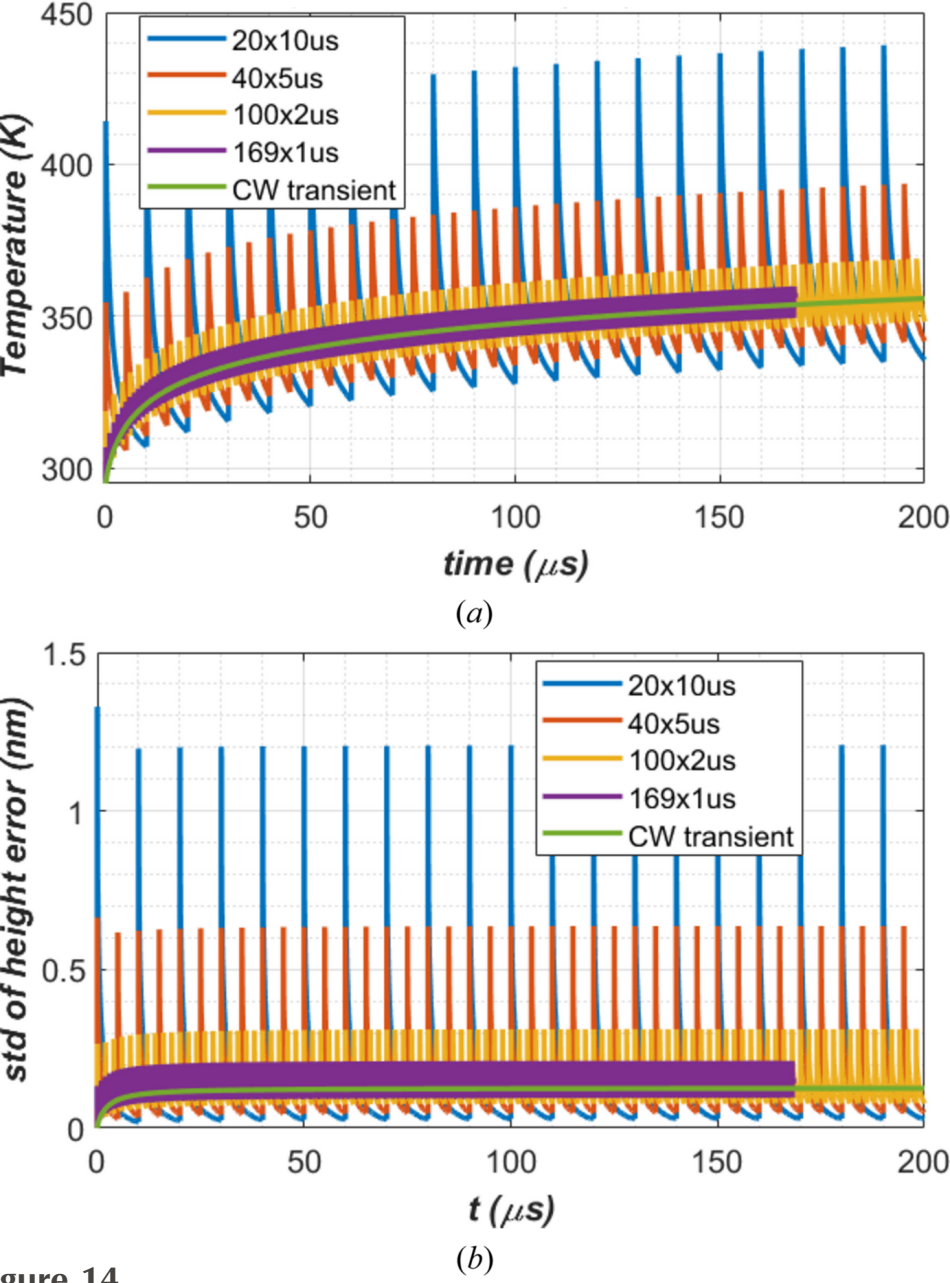
(*a*) The temperature at the center of footprint, (*b*) standard deviation of the height error of the beam footprint on the diamond crystal over 2 × FWHM length. CW and four different repetition rates or repetition times *t*_per_ = 1, 2, 5 and 10 µs with the same average power 262 W, and pulse energy at 0.262, 0.524, 1.31 and 2.62 mJ, respectively. The legends are in the format of *Nt*_per_, where *N* is the number of pulses, except for the CW case.

FEA results for the temperature at the center of the footprint (*T*_0_) and the standard deviation of the height error (*z*_std_) over twice the FWHM length of the beam footprint on the diamond crystal are shown versus time in Fig. 14 ▸ for the cases of CW transient and *t*_per_ = 1, 2, 5 and 10 µs. After the first 200 µs of FEL exposure, the temperature of the diamond crystal [Fig. 14 ▸(*a*)] has not yet reached quasi steady-state. But the thermal deformation [Fig. 14 ▸(*b*)] reaches quasi steady-state in about 50 µs. In general, both temperature and thermal deformation in height error for any repetition time *t*_per_ vary with time around the results for the CW case. The variation amplitude increases with repetition times *t*_per_ or pulse energy *Q*_p_. Both peaks and valleys in the case of lower repetition time *t*_per_ are closer to the results for the CW case than in the case of a longer repetition time *t*_per_. The CW case can be considered as the repetition time tends to zero, or repetition frequency tends to infinity. Therefore, the results from the CW case are upper bounds of the valleys in both temperature and thermal deformation.

**Figure 15:**
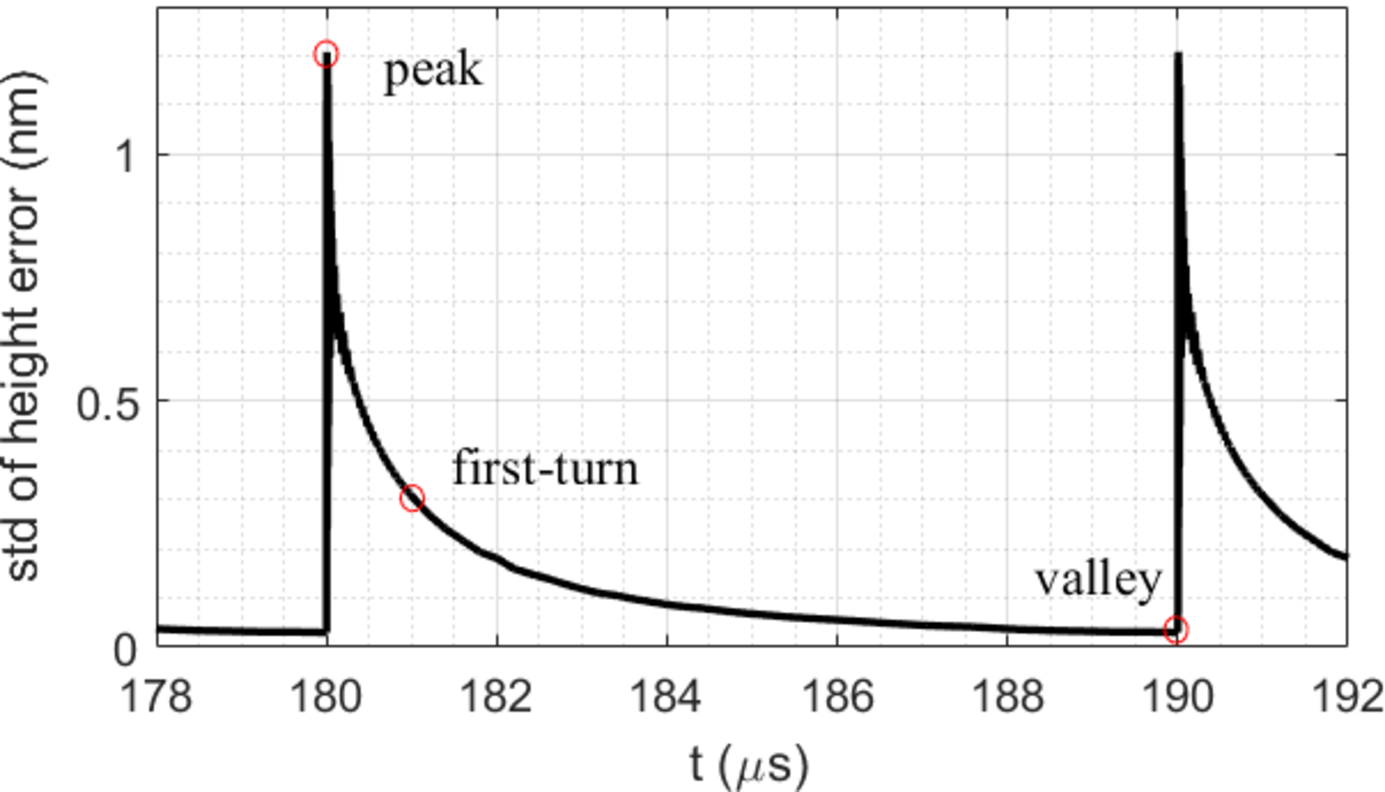
One period of the standard deviation of the height error over the footprint versus time for the case of *t*_per_ = 10 µs. Peak, first-turn and valley points on the curve are indicated.

During each period, there are two crucial times at which the shape of the crystal matters in terms of optical quality: (1) the period-end time (*t*_per-end_ = *it*_per_), the time just before the arrival of the subsequent FEL pulse; this is also the time at which the temperature and thermal deformation reach valleys; and (2) the first-turn time for the CBXFEL as mentioned in the *Introduction*[Sec sec1]. For a 300 m-long CBXFEL cavity, this time is equal to *t*_cavity_ = 1 µs after the FEL pulse. For the *i*th FEL pulse, the first-turn time is equal to (*i* − 1)*t*_per_ + *t*_cavity_. One period of the standard deviation of the height error over the footprint is plotted in Fig. 15 ▸ for the case of *t*_per_ = 10 µs. The points corresponding to the peak, first-turn and valley are indicated on the curve. The peak is reached in a short time after the end of the pulse.

**Figure 16:**
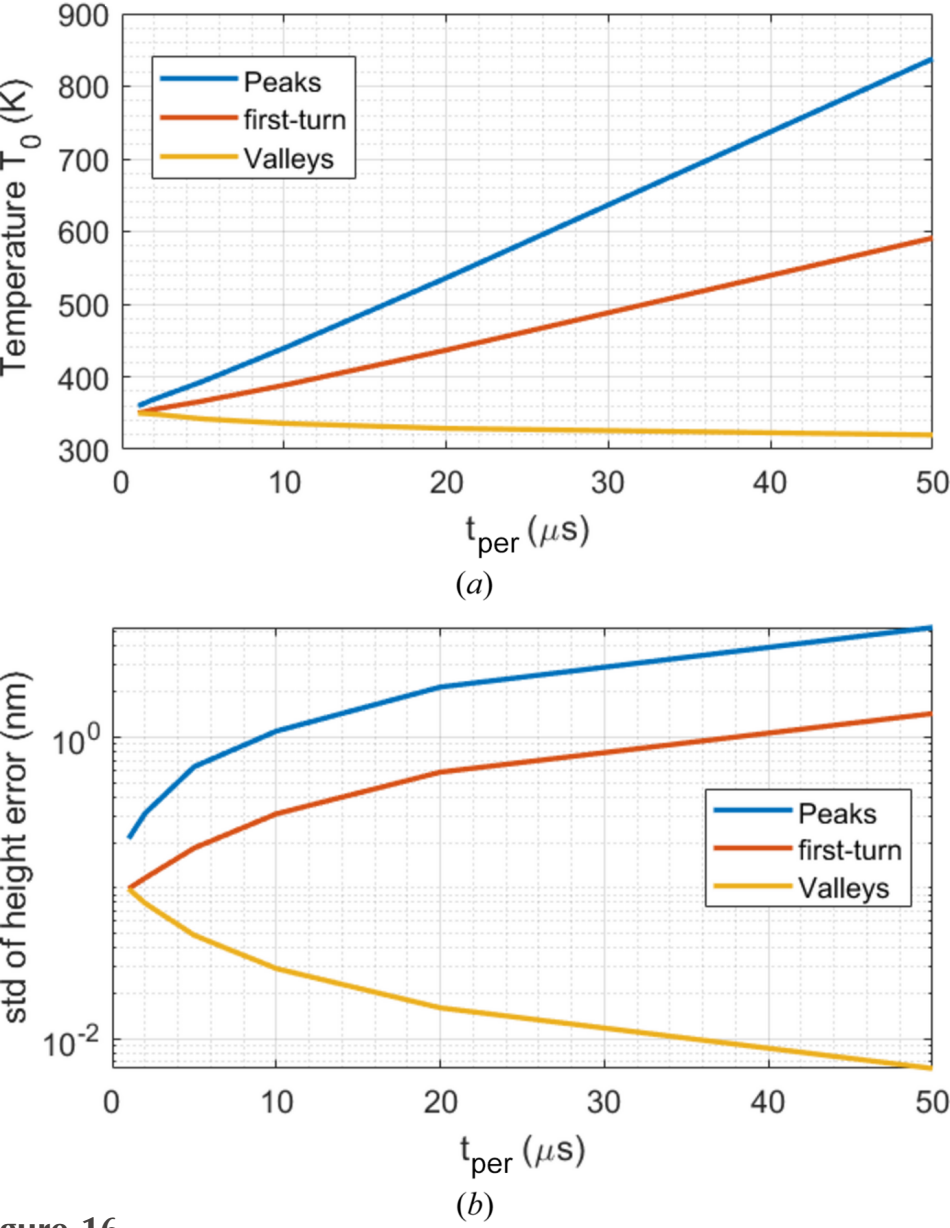
Peaks, first-turn time and valleys during the last period towards 200 µs versus repetition time for (*a*) temperature at the center of the beam footprint *T*_0_ and (*b*) standard deviation height error *Uz*_std_. The same average power of 262 W implies the pulse energy *Q*_p_ is proportional to the repetition time *t*_per_.

From the results shown in Fig. 14 ▸, we take the data of the peak, of the first-turn time and of the valley during the last period towards to 200 µs, shown in Fig. 16 ▸ versus repetition time. Temperatures and thermal deformation at the peaks and the 1 µs first-turn time increase with repetition time or pulse energy, but for the valleys decrease with repetition time or pulse energy. The amplitude of the peaks just after the FEL pulse increase nearly proportionally to the pulse energy. For the case of *t*_per_ = 1 µs, the first-turn and valley in both temperature and height error are identical.

**Figure 17:**
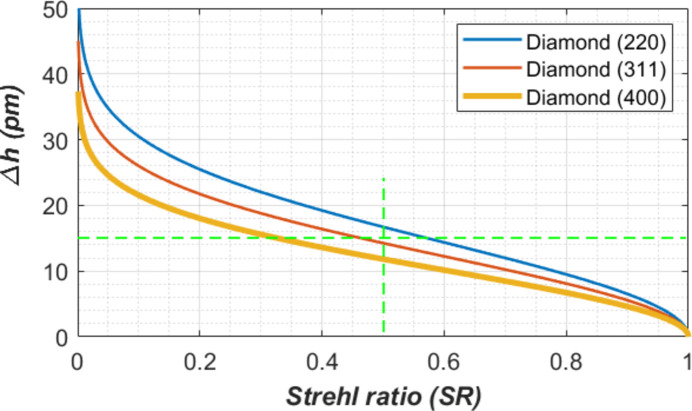
The requirement for wavefront preservation in RMS figure error versus Strehl ratio for diamond crystals. The green dashed lines are a guide to the eye.

The requirement in RMS figure error of a monochromator crystal plane for wavefront preservation was discussed by Zhang *et al.* (2023 ▸). For the (400), (220) and (311) reflections from diamond the requirements in figure error are plotted in Fig. 17 ▸. For diamond (400), the figure error should be smaller than 12 pm to reach a Strehl ratio of 0.5. The standard deviation of the height error (*Uz*_std_) of the diamond crystal at the period-end time just before the arrival of the subsequent FEL pulse [Fig. 15 ▸(*b*)] is about 16 pm for *t*_per_ = 20 µs or 50 kHz repetition rate and pulse energy of 5.24 mJ. The corresponding Strehl ratio is about 0.35. With the repetition rate at 100 kHz and average power of 262 W, the height error of the crystal reaches 30 pm, and the Strehl ratio drops to 0.01. Here the diamond crystal is water cooled and no second-order shape correction was applied. For an average power greater than 262 W and a repetition rate greater than 50 kHz, second-order corrections by focusing optics and/or cryo-cooling become necessary to maintain the optical properties of the diamond crystal.

Note that in this study we assumed constant material properties for the diamond crystal. In that case the temperature rise and thermal deformation in slope error and height error are proportional to the FEL pulse energy.

**Figure 18:**
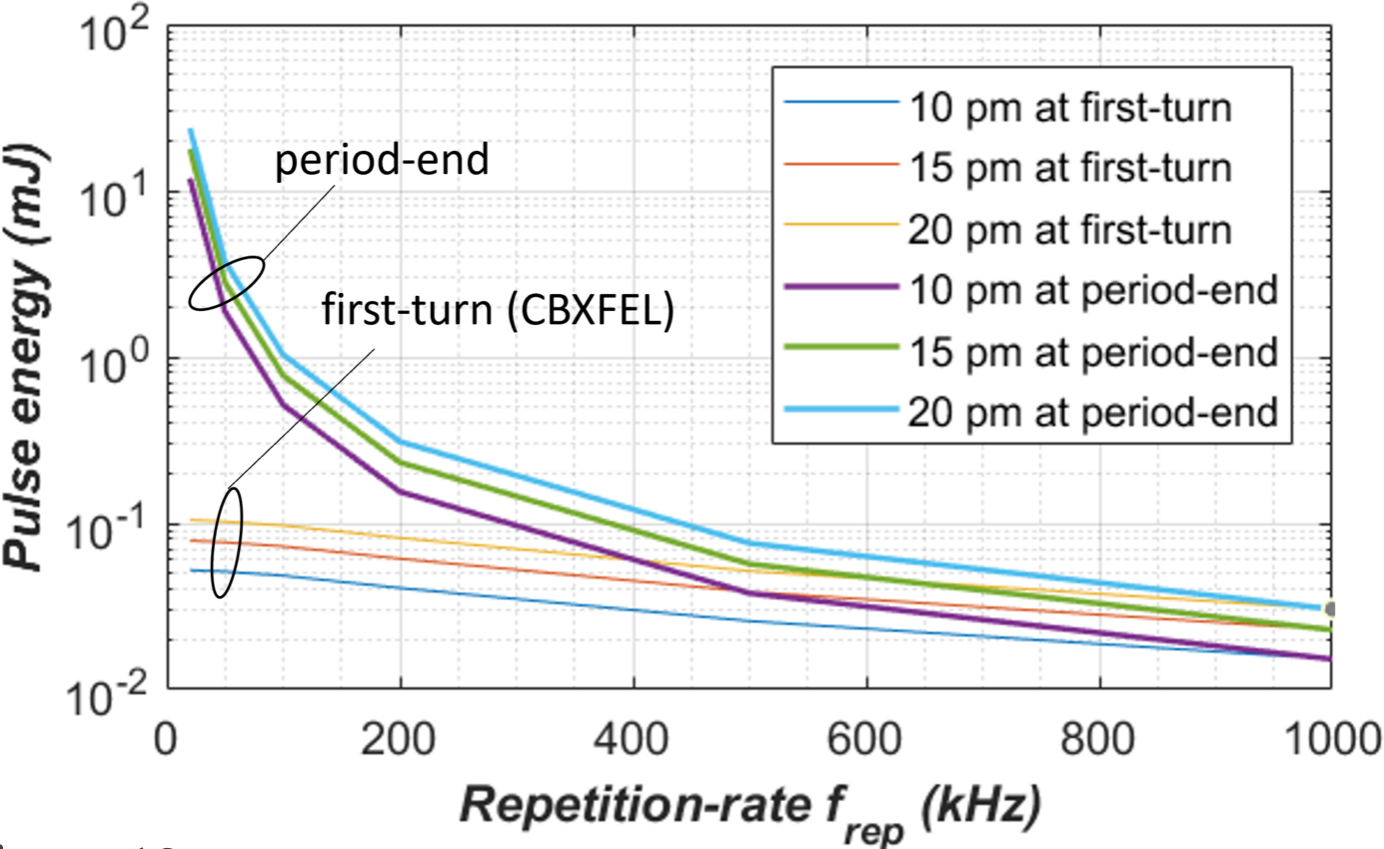
The pulse-energy and repetition-rate phase space to define acceptable operational parameters for the wavefront preservation requirement of the CBXFEL diamond crystal optics.

For the wavefront preservation requirement, the height error of the crystal should be smaller than, for instance, Δ*h* = 10, 15 and 20 pm. For the first crystal C_1_ in the CBXFEL cavity, the upper bound of the pulse energy *Q*_p_ corresponding to Δ*h* can be then determined as follows,
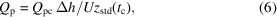
where *Uz*_std_(*t*_c_) is shown in Fig. 16 ▸(*b*) for valleys at the period-end time or at first-turn time, and *Q*_pc_ is the pulse energy used in the calculation and corresponds to 262 W average power as *Q*_pc_ = 0.262*t*_per_ where the units of *Q*_pc_ and *t*_per_ are, respectively, mJ and µs. Equation (6) defines a pulse-energy repetition-rate phase space. Results are shown in Fig. 18 ▸ for both the thermal deformation at the first-turn time and at the period-end time *t*_per-end_. The pulse energy upper limit at both critical times (first-turn, period-end) decreases with repetition rate, especially at the period-end time. For the wavefront preservation requirement of the CBXFEL diamond crystal optics, the operational parameters such as pulse-energy and repetition-rate should be chosen in the space below the curves. The single shot damage on the crystal optics should also be considered in the definition of the operational pulse energy.

From results shown in Fig. 18 ▸, the pulse energy for CBXFEL diamond crystal with an RMS height error of less than 20 pm at 100 kHz should be limited to 0.1 mJ, instead of 2.62 mJ. The temperature at the center of the beam footprint on the diamond crystal surface shown in Fig. 16 ▸(*a*) at 2.62 mJ and 100 kHz (10 µs repetition time) is 439.2 K just after the FEL pulse, 388.2 K at the 1 µs first-turn time, and 335.6 K at the 10 µs repetition time. As the pulse energy should be limited to 0.1 mJ for the CBXFEL crystal, the maximum temperature in the crystal at the first-turn time is then (388.2 − 295) × 0.1/2.62 + 295 = 298.6 K, which is just 3.6 K of temperature increase relative to the initial uniform temperature of 295 K.

## 6. Summary and conclusion

In this paper, we have studied transient thermal deformation of a diamond crystal for high-repetition-rate XFELs, looking toward a CBXFEL, by numerical simulations including FEA and advanced post data processing. Pulse-by-pulse transient thermal deformation of a 50 µm-thick water-cooled diamond crystal has been performed with X-ray pulse repetition rates between 50 kHz and 1 MHz. Full transient temperature and thermal deformation of the crystal have been compared with the results of a transient analysis in the CW case. Both temperature and thermal deformation at any repetition times *t*_per_ or repetition rate *f*_rep_ vary with time about the results for the CW case. The variation amplitude increases with repetition time, *t*_per_, or pulse energy, *Q*_p_. Both the peaks and valleys are getting closer and closer to the results for the CW case when the repetition time *t*_per_ become shorter and shorter (repetition rate *f*_rep_ becomes larger and larger). In fact, when the repetition rate tends to infinity (repetition time tends to zero), both temperature and thermal deformation converge to the results for the CW case. Two critical times for acceptable operation of the crystal are (1) the first-turn time, *i.e.* the time for the XFEL pulse to complete the first turn around the cavity so that the crystal sees the recirculated XFEL pulse; and (2) the period-end time, *i.e.* the time that the next electron bunch arrives for the amplification, so that the crystal outcouples the amplified FEL power. The period-end time is critical for both a CBXFEL cavity and the X-ray optics for beam transport in the beamline. For the same average power, simulation results show that the crystal thermal deformation seen by the XFEL beam decreases with repetition rate at the first-turn time of a 300 m-long cavity but increases with repetition rate at the period-end time. For the wavefront preservation requirement of the crystal, a pulse-energy repetition-rate phase space has been established. This can be used to define acceptable operating parameter ranges such as pulse energy and repetition rate. The pulse energy upper limit at both critical times (first-turn and period-end) decreases with repetition rate, especially at the period-end time.

For instance, to reach an RMS height error of less than 20 pm in the diamond crystal, (1) the pulse energy should be limited to less than 100 µJ at 100 kHz repetition rate and 30 µJ at 1000 kHz repetition rate for a 300 m-long CBXFEL (see curves for first-turn in Fig. 18 ▸), and (2) for a beam transport optics (see curves for period-end in Fig. 18 ▸) these limitations can be 1000 µJ at 100 kHz repetition rate, 30 µJ at 1000 kHz repetition rate.

The upper bound of the thermal deformation of the crystal at the period-end time for any repetition frequency can be estimated from the CW case. For average power higher than 262 W and repetition rate greater than 50 kHz, second-order correction by focusing optics and/or cryo-cooling become necessary for the performance of this diamond crystal.

To ensure the accuracy of the simulations and reliability of the simulation results, we have investigated or optimized parameters related to the FEA, such as mesh size, time increment, power loading pulse length and damping ratio. The time durations to reach steady state or quasi steady-state for both temperature and thermal deformation from FEA has been compared with analytical estimates and are in good agreement. For a 5 mm × 5 mm × 0.05 mm diamond crystal, the time to reach quasi steady-state is about 50 ms for temperature and 50 µs for thermal deformation.

## Figures and Tables

**Table 1:** Material properties of the diamond crystal

Density (kg m^−3^)	Young’s modulus (GPa)	Poisson’s ratio	Thermal conductivity (W m^−1^ K^−1^)	Thermal expansion	Heat capacity (J kg^−1^ K^−1^)
3520	1000	0.1	1900	10^−6^	520
